# Contraceptive use and contraceptive counselling interventions for women of reproductive age with cancer: a systematic review and meta-analysis

**DOI:** 10.1186/s12916-022-02690-w

**Published:** 2022-12-17

**Authors:** Melissa L. Harris, Tesfaye R. Feyissa, Nikola A. Bowden, Kristina Gemzell-Danielsson, Deborah Loxton

**Affiliations:** 1 Centre for Women’s Health Research, College of Health, Medicine and Wellbeing, University of Newcastle, Newcastle, New South Wales Australia; 2 Hunter Medical Research Institute, Newcastle, New South Wales Australia; 3 School of Medicine, Faculty of Health, Deakin University, Geelong, Australia; 4 Centre for Drug Repurposing and Medicines Research, College of Health, Medicine and Wellbeing, University of Newcastle, Newcastle, New South Wales Australia; 5 Department of Women’s and Children’s Health, Karolinska Institutet and Karolinska University Hospital, Stockholm, Sweden

**Keywords:** Cancer, Contraception, Contraceptive counselling, Women, Interventions

## Abstract

**Background:**

A lack of clarity exists regarding contraceptive uptake and counselling among women with cancer, despite these women having unique family planning needs. This study aimed to systematically review the available literature and produce an overall summary estimate of contraceptive use and counselling among women with cancer across the cancer care continuum.

**Methods:**

A systematic search of articles reporting on contraceptive counselling and/or contraceptive use among women of reproductive age (15–49 years) with cancer across the cancer care continuum (e.g. diagnosis, treatment, survivorship) was conducted in MEDLINE, Embase, CINAHL, Maternity and Infant Care and Cochrane Library. Two independent reviewers conducted the data screening, data extraction and risk of bias assessment. Qualitative synthesis and meta-analyses were conducted to summarise the key findings.

**Results:**

We included 21 articles involving 3835 participants in this review. Studies varied according to the cancer population and time along the cancer care continuum it was assessed. Of the studies that reported the overall contraceptive prevalence among women diagnosed with cancer (*n* = 8), contraceptive use ranged from 25 to 92%. Of the four studies that focused on cancer survivors, the contraceptive prevalence ranged from 47 to 84%. When the prevalence of these studies was pooled, a crude summary prevalence of 64% (62% among women with cancer versus 68% among cancer survivors) was found. The rate of contraceptive counselling was assessed in ten studies. A pooled prevalence of 50% (44% among women with cancer versus 58% among cancer survivors) was found, with the prevalence ranging from 12 to 78% among individual studies depending on the point in the cancer care continuum that it was provided. When contraceptive counselling was provided, it was found to significantly increase contraceptive use although biases were identified in its application.

**Conclusions:**

Contraceptive counselling interventions as part of standard cancer care have the potential to not only empower women with cancer and cancer survivors to make informed choices regarding their reproductive health but also provide the ability to plan future pregnancies for times of better health.

**Supplementary Information:**

The online version contains supplementary material available at 10.1186/s12916-022-02690-w.

## Background

The global burden of cancer is increasing, particularly among women. Cancer has one of the highest mortality rates among high-development index countries, and breast cancer is the most diagnosed type worldwide [1]. Among women impacted by cancer, up to a third of cases have been found to occur during the reproductive years [1, 2]. Women diagnosed with cancer at this life stage have unique family planning needs, particularly given the increasing numbers of women being diagnosed before they have started or completed childbearing [3]. Currently, there is recognition of, and recommendations around, the receipt of time-sensitive fertility counselling for young women (particularly those diagnosed and treated in childhood or adolescence) [4–7]. Some of these guidelines (e.g. European Society of Medical Oncology guidelines) highlight the general need for effective contraception in the context of systemic anticancer treatment. However, there are no guidelines or clinically endorsed physician resources regarding tailored contraception recommendations for specific cancer types during or after cancer treatment [8, 9]. This represents a critical gap in patient care as most women retain their fertility potential following cancer treatment, with the absolute risk of infertility remaining low (around 8% for childhood cancer survivors), even in the presence of gonadotoxic therapy [10, 11]. As such, the gap in the provision of contraceptive care for women who have experienced cancer has been associated with higher rates of unintended pregnancy and medical abortion compared to other women [12, 13].

It has been recommended that women undergoing cancer treatment or adjuvant therapy including surgery, radiotherapy and chemotherapy as well as hormonal and targeted therapies avoid pregnancy due to adverse maternal and foetal impacts [2, 14, 15]. With most partnered women (83%) remaining sexually active during and after cancer treatment, contraceptive counselling should be an essential component of the treatment journey [16, 17]. This is particularly important as the pill is the most popular contraceptive method across the reproductive life course, but avoidance of oestrogen and progestogen-containing hormones (present in the combined oral contraceptive pill) is recommended for women with specific cancer types [18]. For women with a history of hormonally mediated cancer (e.g. breast and endometrial cancers) and women who have received thoracic radiation, it is argued that non-hormonal contraceptive methods should be considered as first-line approaches, due to the potential impacts on prognosis and cancer recurrence [19]. In contrast, women who are at high risk of developing breast and ovarian cancers (i.e. have BRCA1 and BRCA2 mutations) could benefit from the use of combined hormonal contraception [20]. A 2013 meta-analysis found that the use of the combined oral contraceptive pill reduced the risk of developing ovarian cancer by around 42%, and a non-significant association was found for breast cancer [21]. In addition, medical eligibility guidelines generally recommend the use of higher effectiveness contraceptive methods such as long-acting reversible contraception [LARC] over lower efficacy methods such as the pill where possible, due to their increased ability to prevent pregnancy [22]. As such, the receipt of time-sensitive contraceptive counselling to identify and facilitate access to contraceptive methods that not only take into account cancer type and stage along the cancer care continuum, but are also tailored to women’s reproductive goals, is of paramount importance [23]. This may be critical to reducing unintended pregnancy risks and ensuring that pregnancies among cancer populations are planned for times of better health [24].

Therefore, contraceptive counselling is as important as fertility preservation for women with cancer, and strategies that improve effective contraceptive uptake among these women are needed. However, there is some evidence to suggest that the provision of contraceptive counselling is suboptimal [25, 26]. In addition, there is a lack of clarity regarding the effectiveness of current contraceptive counselling interventions and their role in increasing the uptake of tailored contraception (including LARC) [27]. Given there are limited studies on the topic, there is a high need for a systematic review to assist with the development and delivery of effective contraceptive counselling programmes. Therefore, the aims of the review are twofold: (1) summarise and describe the prevalence and efficacy of contraceptive use among women with cancer and (2) evaluate contraceptive counselling practice and its effectiveness in improving the uptake of effective contraception among women with cancer.

## Methods

This systematic review and meta-analysis conformed to the Preferred Reporting Items for Systematic Reviews and Meta-Analysis guidelines (PRISMA) 2020 [28]. The protocol for the review was registered with PROSPERO (number CRD42021278322).

### Eligibility criteria

Human studies written in English and reporting on contraceptive counselling and/or contraceptive use among women of reproductive age (15–49 years) with cancer across the cancer care continuum (e.g. diagnosis, treatment, survivorship) were eligible for inclusion [22]. Original research or peer-reviewed conference abstracts using qualitative, cross-sectional, case-control, cohort, quasi-experimental, non-randomised intervention or randomised controlled trial (RCT) study designs were considered. Given that the interest in the contraceptive practices of women with cancer is an emerging area of concern, no limits to study time frames and geographic locations were included.

Case reports, clinical guidelines, position papers and papers focused on clinician perspectives of contraceptive counselling were excluded. Studies with ambiguous outcomes in which the measurement of contraception was unclear and studies that included men (where data for women could not be disaggregated) were also ineligible. While any form of contraceptive counselling provided by a trained health care provider (e.g. oncologist, gynaecologist or nurse) was considered, if the timing of counselling occurred before a cancer diagnosis only, the study was excluded. Furthermore, studies focused on the relationship between contraceptive use (e.g. the combined oral contraceptive pill) and cancer onset were also ineligible. If a peer-reviewed abstract and full paper from the same study was found, we retained the peer-reviewed paper for review. A detailed summary of the inclusion and exclusion criteria is shown in Additional file 1: Table S1.

### Data sources and search strategy

A systematic search of five databases including MEDLINE, Embase, Cumulative Index to Nursing and Allied Health Literature (CINAHL), Maternity and Infant Care and Cochrane Library was undertaken during August and September 2021. The electronic database search strategy was initially developed by TRF using MEDLINE MeSH terms. The search strategy was reviewed by MLH and then refined with the assistance of the College of Health, Medicine and Wellbeing Librarian. The search strategy covered the concepts of (1) contraceptive use and contraceptive counselling and (2) cancer. This search strategy was then expanded to other databases. Google Scholar was used to identify additional articles from bibliographies of the selected articles and studies citing the articles. The search strategy for Ovid MEDLINE is shown in Additional file 1: Table S2.

### Selection process

Two reviewers (TRF and MLH) independently conducted all stages of the selection process. Studies were downloaded into the Covidence online software, and duplicates were removed. Articles were screened for eligibility based on their title and abstracts according to the inclusion and exclusion criteria. Full-text articles (where applicable) were retrieved and screened for relevancy. If a peer-reviewed journal abstract was eligible, the whole article was read, and the abstract was excluded. Where necessary, the authors were contacted to fill in the missing information. Disagreements were resolved through a discussion between the two reviewers.

### Data collection process and data items

Data on the included studies were independently extracted by the first and second authors (MLH and TRF). The data extraction sheet was adapted from the Cochrane checklist of items for data collection and contained information on the characteristics of the study (name of the first author and year, country, study design and settings), participant characteristics (age, total participants), cancer type and treatment, intervention characteristics (presence of contraceptive counselling and source), key findings (contraceptive use and change including specific methods) and study limitations [29]. A large number of articles reported contraceptive use according to the World Health Organization’s tiered efficacy criteria (tiers I to IV) (see Table 1) [30, 31]. Where detail was reported on individual contraceptive methods, this level of data was also extracted. For studies reporting on contraceptive counselling interventions, we detailed how the counselling was delivered, when (i.e. before or after cancer diagnosis), by whom and for how long. Data extractions were conducted from September to October 2021.

**Table 1:** World Health Organization-tiered approach to contraceptive effectiveness [30]

Tier	Typical use failure rate	Contraceptive method
I: failure rate < 1% per year	0.05	Implant
	0.2	Levonorgestrel intrauterine system
	0.8	Copper intrauterine device
	0.15	Male sterilisation
	0.5	Female sterilisation
II: failure rate 6–12% a year	6	Depot-medroxyprogesterone acetate injection
	9	Combined oral contraceptive pill and the progestogen-only pill
	9	Contraceptive patch
	9	Contraceptive ring
	12	Diaphragm
III: failure rate 18–24% per year	18	Male condom
	21	Female condom
	22	Withdrawal
	24	^a^Sponge (parous women)
IV: failure rate ≥ 24% per year	24	Fertility awareness methods
	28	Spermicide

The WHO criteria are based on the percentage of women who will become pregnant within the first year of typical contraceptive use of the method. Under typical use conditions, tier 1 methods (e.g. the levonorgestrel intrauterine device [IUD]) have annual failure rates of < 1% while tier II (e.g. the combined oral contraceptive pill) methods have failure rates of 6–12%. Tier III (e.g. condoms) have failure rates between 18 and 21%, and tier IV methods (e.g. fertility-based awareness methods) have failure rates in excess of 24% per year

^a^Sponge failure rate among nulliparous women is 12%

### Risk of bias assessment of observational studies

For the risk of bias assessment of the cross-sectional and cohort studies, the Joanna Briggs Institute (JBI) risk of bias assessment tool was used [32]. This tool focused on three components: design, conduct and analysis. Two reviewers (TRF and MLH) rated each of the nine items independently according to the dichotomous ratings: yes and no/unclear. Any disagreements were resolved through a discussion. Studies with a higher score indicated a lower risk of methodological bias. Risk of bias assessment based on these scores was then categorised into high risk of bias (1–3), medium risk of bias (4–6) and low risk of bias (7–9).

### Synthesis methods and effect measures

Qualitative synthesis was used to summarise the key findings. Where possible, odds ratios (ORs) and relative risks (RRs) related to contraceptive counselling interventions or contraceptive use were reported. The synthesis mechanism depended on the type of cancer and contraceptive counselling. Structured approaches were used by tabulating and coding the main characteristics of women with cancer, contraceptive counselling interventions and contraceptive use. The overall prevalence of contraceptive use among women with cancer (during or after treatment) was summarised. Where possible, specific contraceptive methods used and method change by women with cancer were also identified. Contraceptive counselling, the sources of counselling and the impact of contraceptive counselling on contraceptive use were also reported when available.

In addition, a summary prevalence estimate of contraceptive use and contraceptive counselling among women with cancer and cancer survivors was calculated by pooling the study-specific estimates. Random effects meta-analysis that considered heterogeneity between studies was conducted due to disparities in designs and participants. Chi-square (*χ*^2^) tests and the *I*^2^ statistic were used to assess the heterogeneity between studies using Stata 17. A score of > 75% was used to indicate heterogeneity between studies. Subgroup analyses were also conducted to investigate the sources of heterogeneity (for women with cancer versus cancer survivors).

## Results

### Study characteristics

**Fig. 1:**
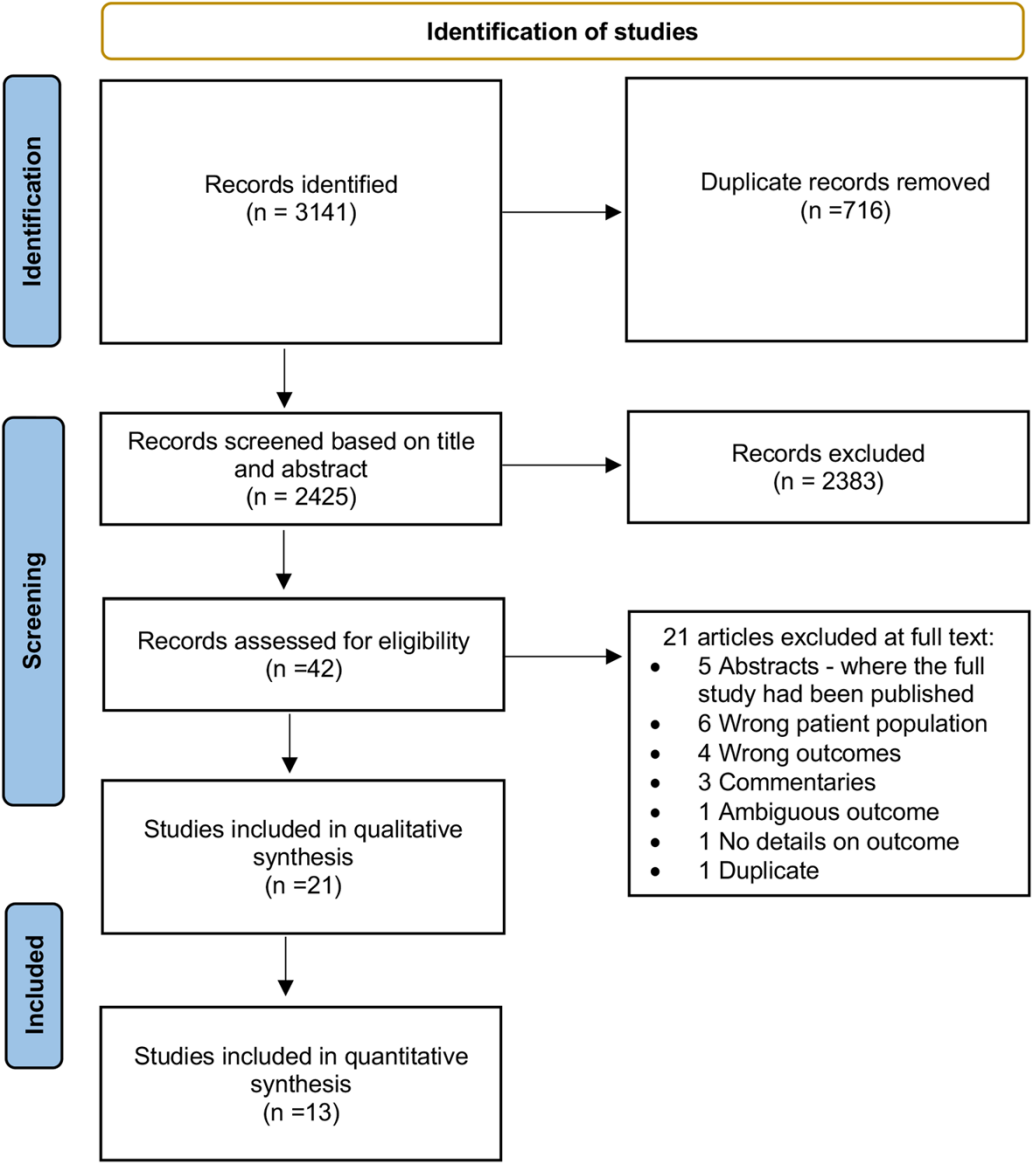
PRISMA study flow diagram

A total of 3141 articles were identified across the five databases. Of these, 716 articles were duplicates, 2383 articles were excluded through the title and abstract screening process and a further 21 articles were excluded after full-text review (Fig. 1). A total of 21 studies published between 2009 [33] and 2021 [34] (involving 3835 participants) were included in this review; 16 of which were peer-reviewed articles, and five of which were peer-reviewed abstracts. More than half of the studies were conducted in the USA (*n* = 12) and involved either cross-sectional self-report surveys (*n* = 9) or retrospective chart reviews (*n* = 5) (Additional file 1: Table S3). Only three studies analysed cohort study data (including one pilot study), two studies involved qualitative interviews and the remaining two studies involved an RCT and pilot intervention. Fifteen studies focused on women with cancer undergoing treatment while six were among reproductive-aged cancer survivors. Breast cancer was the most common type of cancer examined (*n* = 20) with nine studies focused exclusively on this cancer type. The type of cancer treatment experienced was reported in 16 studies. These primarily included surgery, chemotherapy, radiation and hormone therapy. In terms of study bias, six observational studies were determined to be at high risk of bias while eight studies were at medium risk of bias (Table 2).

**Table 2:** Risk of bias assessment of observational studies

Authors	Item 1	Item 2	Item 3	Item 4	Item 5	Item 6	Item 7	Item 8	Item 9	Total
Abelman et al. 2020 [25]	N	Y	N	Y	Y	Y	Y	N	N	5
Castro-Sanchez et al. 2018 [35]	N	Y	N	Y	Y	Y	Y	N	N	5
Cutler et al. 2016 [36]	N	Y	N	Y	Y	N	Y	N	N	4
Dominick et al. 2015 [18]	Y	Y	N	Y	Y	Y	Y	Y	Y	8
Franca et al. [37]	N	N	N	N	Y	Y	Y	N	N	3
Guth et al. 2016 [38]	N	Y	N	Y	Y	Y	Y	N	N	5
Hadnott et al. 2019 [23]	Y	Y	Y	Y	Y	Y	Y	N	N	7
Johansen et al. 2017 [39]	N	Y	N	Y	Y	Y	Y	N	N	5
Knight et al. 2014 [40]	N	N	N	N	N	Y	Y	N	N	2
Lakhdissi et al. 2017 [41]	N	N	N	N	N	Y	Y	N	N	2
Madrigal et al. 2019 [42]	N	N	N	Y	Y	Y	Y	N	N	4
Maslow et al. 2014 [43]	N	Y	N	Y	Y	Y	Y	Y	Y	7
Massarotti et al. 2021 [34]	N	Y	N	N	Y	Y	Y	N	N	4
McLean et al. 2014 [44]	N	Y	N	Y	N	N	N	Y	N	3
Mody et al. 2019 [26]	Y	Y	N	Y	Y	Y	Y	N	N	6
Patel et al. 2015 [45]	N	N	N	Y	Y	Y	N	N	N	3
Patel et al. 2009 [33]	N	N	N	Y	Y	Y	N	N	N	3
Quinn et al. 2014 [46]	Y	Y	Y	Y	Y	Y	Y	N	N	7

Item 1: Was the sample representative of the target population?

Item 2: Were the study participants recruited in an appropriate way?

Item 3: Was the sample size adequate?

Item 4: Were the study subjects and the setting described in detail?

Item 5: Was the data analysis conducted with sufficient coverage of the identified sample?

Item 6: Were objective, standard criteria used for the measurement of the condition?

Item 7: Was the condition measured reliably?

Item 8: Was there an appropriate statistical analysis?

Item 9: Are all important confounding factors/subgroups identified and accounted?

### Contraceptive use among women with cancer and cancer survivors

The prevalence of contraceptive use varied according to the time point it was provided along the cancer care continuum. Half of the studies reported contraceptive use at either diagnosis or during treatment (*n* = 8) or in survivorship (*n* = 4). Only one study examined contraceptive use prior to, during treatment and post-treatment [26]. Of the studies that reported the overall contraceptive prevalence among women diagnosed with cancer (*n* = 8), contraceptive use ranged from 25 [39] to 92% [47]. Of the four studies that focused on cancer survivors, the contraceptive prevalence ranged from 46.6 [46] to 84% [23]. Only one study evaluated the emergency contraception use among cancer survivors (one-third of which included breast cancer survivors) [44]. There was a lack of high-quality studies with only one RCT [47] and one prospective cohort study [18] included.

**Fig. 2:**
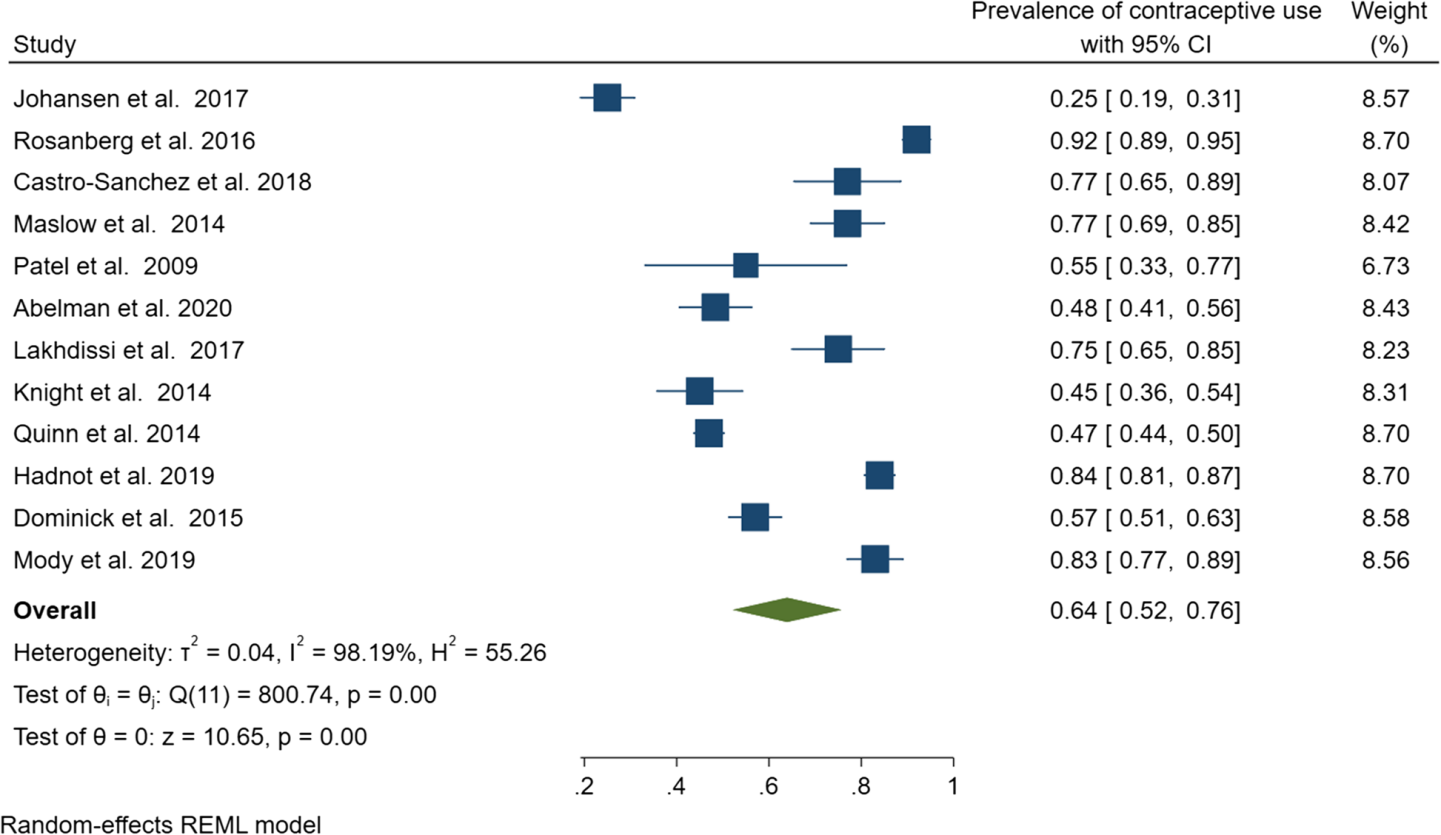
Pooled estimate of contraceptive use among women with cancer and cancer survivors

**Fig. 3:**
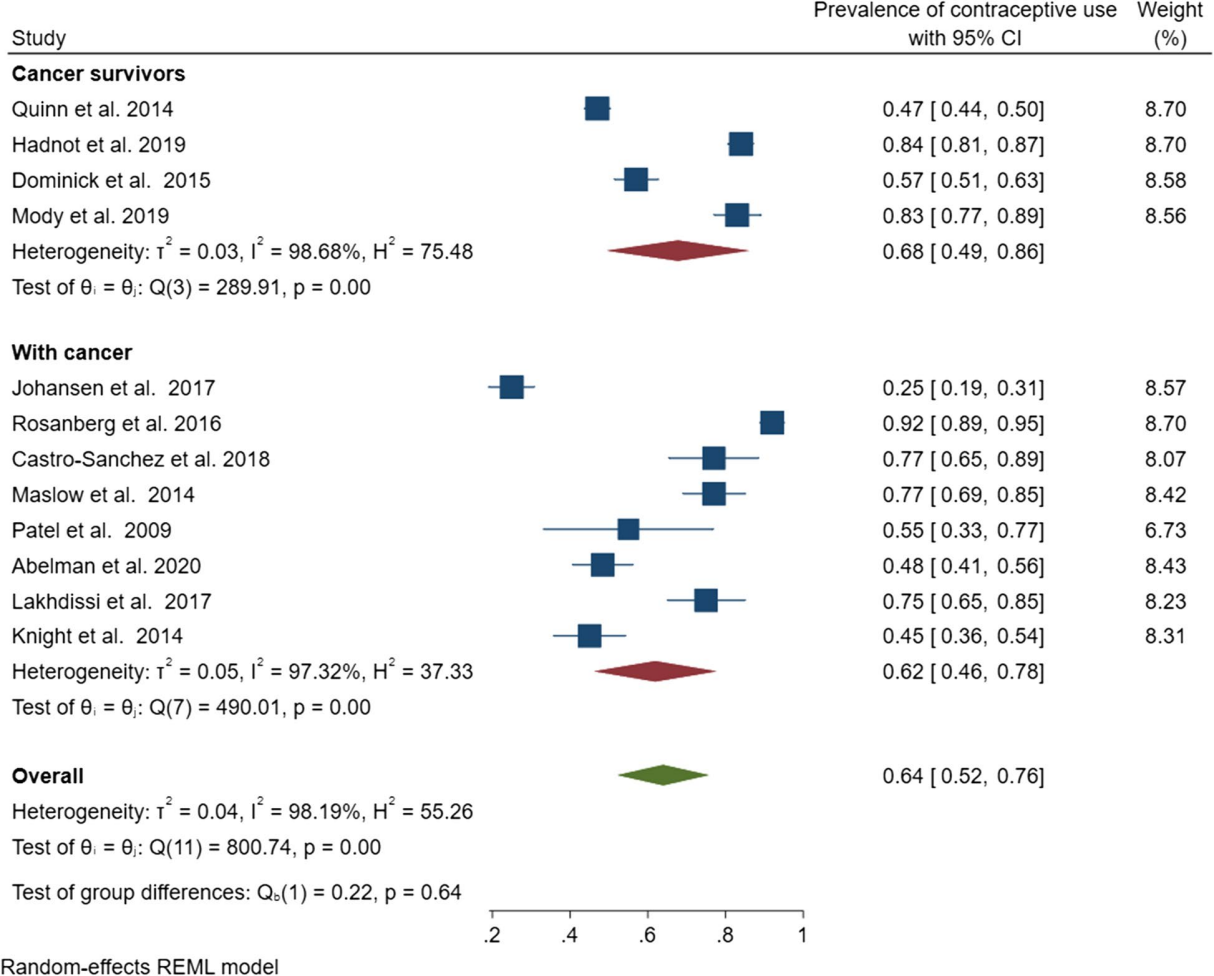
Subgroup analysis of contraceptive use by cancer status (women with cancer versus cancer survivors)

The pooled contraceptive prevalence among women with cancer and cancer survivors was 64% (95% CI: 52–76%); however, significant evidence of heterogeneity was identified (*Q* = 800.74, *r*^2^ = 0.04, *I*^2^ = 98.2%, *p* < 0.001) (Fig. 2). Subgroup analyses of contraceptive use by cancer status produced a pooled prevalence of 62% (95% CI: 46–78%) (*Q* = 490.01, *I*^2^ = 97.3%, *p* < 0.001) among women with cancer and 68% (95% CI: 49–86%) (*Q* = 289.91, *I*^2^ = 98.7%, *p* < 0.001) among cancer survivors (Fig. 3). Given the studies were highly heterogeneous, a narrative review describing the individual studies was warranted.

### Efficacy of contraception used by women with cancer

Among the studies that focused on contraceptive use among women undergoing treatment for cancer (*n* = 8), an overall increase in the use of less effective methods following diagnosis was found. A Mexican cross-sectional study (*n* = 104) found 51% of reproductive-aged women with breast cancer were users of contraception during chemotherapy treatment, and 46% used contraception while receiving long-term hormone therapy/trastuzumab. Only 29% of those who reported being sexually active used effective tier I contraceptive methods. Also, one woman was still found to be using a hormonal-based contraceptive method during chemotherapy and while receiving long-term adjuvant treatment [35]. A retrospective chart review showed that 45% of women had documentation of contraceptive methods prior to initiation of treatment [40]. Lower use of effective tier I/II methods (27%) compared with tier III/IV (35%) methods following a cancer diagnosis was also reported in the USA [43]. Specifically, Maslow and colleagues [43] found that 21% of women used condoms, 10% used withdrawal and 21% used the oral contraceptive pill. Only 5% of women with cancer used high efficacy tier I contraception (IUD or partner vasectomy) following diagnosis. On the other hand, a US cross-sectional study reported about half of the women with cancer used barrier methods (condoms and foams), while abstinence was preferred by the remainder of women [33]. In contrast, a pilot intervention study in the USA found the uptake of highly effective contraception to be substantially higher. Among sexually active contraceptive users, more than half were using tier I methods. Only 11.1% used tier II methods, and 36.1% used tier III methods [42]. This finding is supported by a South African qualitative study which reported that about two-thirds of women were contraceptive users, half of which involved the copper IUD [48].

Finally, two studies assessed contraceptive use in very young women with cancer. A retrospective study among women aged 15–25 years with cancer (40.8% with haematological cancers) in the USA showed that only 48.4% had documented contraceptive use during cancer treatment. Specific methods included the combined oral contraceptive pill (24.2%), condoms (7%), depo injection (9.5%), progestogen-only implant (1.3%) and IUD (4.5%). A small proportion of women (1.9%) used other methods such as the progestogen-only oral contraceptive pill, vaginal ring and withdrawal [25]. Among sexually active Brazilian teenage girls with cancer (42% osteosarcoma and 36% leukaemia), 89% used tier II methods such as oral or injectable hormonal contraceptives while 15% used condoms (tier III methods). Importantly, over one-fifth had never used any contraception despite being sexually active [37].

### Contraceptive use among cancer survivors

Contraceptive use among cancer survivors (i.e. post-treatment) was examined in five studies [18, 23, 26, 44, 46]. Overall, cancer survivors were less likely to use contraception compared to the general population, and when contraception was used, it was more likely to be of lower efficacy. In a cross-sectional study of US cancer survivors aged 18–40 years, around one-third were found to be users of highly effective tier I methods (most of which were LARC). The majority of contraceptive users, however, used less effective methods with around one-quarter using tier II methods (primarily the combined oral contraceptive pill), 29% using tier III methods (half using barrier or withdrawal methods) and 11% using tier IV methods [23]. Similar disparities were reported in prospective research. For instance, Dominick et al. found a lower rate of contraceptive use among cancer survivors compared with the general US population (34% vs 53%, *p* < 0.01). Tier II (37% used the combined oral contraceptive pill) and III (36% used condoms) methods were the most reported contraceptive methods with only 13% using a tier I method (IUD). Ten per cent of women were also found to use post-coital emergency contraception [18]. In contrast, a retrospective pilot study of US non-gynaecological cancer survivors (with linkage to the California Cancer Registry) found tier III methods (withdrawal and barrier methods) were the main forms of contraception used, with a further 25% using tier II short-term hormonal methods. When women engaged in tier I methods, it was most likely to be in the form of permanent sterilisation (37%) [46].

### Contraceptive change across the cancer care continuum

Two studies examined contraceptive change following cancer diagnosis, and the findings were inconsistent. A prospective cohort study in Switzerland found that 58% of women were users of low-efficacy contraception following the diagnosis of breast cancer. No contraceptive method, rhythm method or withdrawal was reported by 34% while condom use was reported by 8%. Hormonal contraception was stopped at diagnosis by 16% of women (10% reported using the oral contraceptive pill and 4% levonorgestrel IUD prior to diagnosis) [38]. In contrast, an RCT in the USA testing an education and support intervention found that contraceptive prevalence increased post-cancer diagnosis (39% vs 52%). However, 6% of women with breast cancer still reported using hormonal contraception despite recommendations against these methods. A further 2% reported withdrawal as their only contraceptive method, and 8% reported no contraception [47].

Contraceptive use across the cancer care continuum (i.e. prior to treatment, during treatment and after treatment) was only examined in one US study (*N* = 150). Among women with breast cancer, increases in the change to high-efficacy reversible or permanent methods (tier I methods) were noted during and after cancer treatment. The use of the copper IUD incrementally increased from 3.3% prior to treatment to 23.4% post-treatment, while permanent contraception increased from 6.5% prior to treatment to 16.1% post-treatment. Although an increase in tier III methods was noted across the cancer care continuum, the use varied depending on at which time point it was captured. For instance, while 21.1% of women used condoms prior to treatment initiation, and 29% used condoms after treatment, its use peaked at 52.4% during treatment. A steady increase in tier IV methods was also identified over time, although the greatest increase was found in the transition from diagnosis to treatment initiation (0.8 to 9.5%) [26].

### Contraceptive counselling among women with cancer

The prevalence of contraceptive counselling among women with cancer or cancer survivors was assessed in ten studies. The rate of contraceptive counselling ranged from 12 [36] to 78% depending on the point in the cancer care continuum that it was provided [45]. Contraceptive counselling was provided after diagnosis and during treatment in six studies [25, 26, 43, 45, 46, 49].

**Fig. 4:**
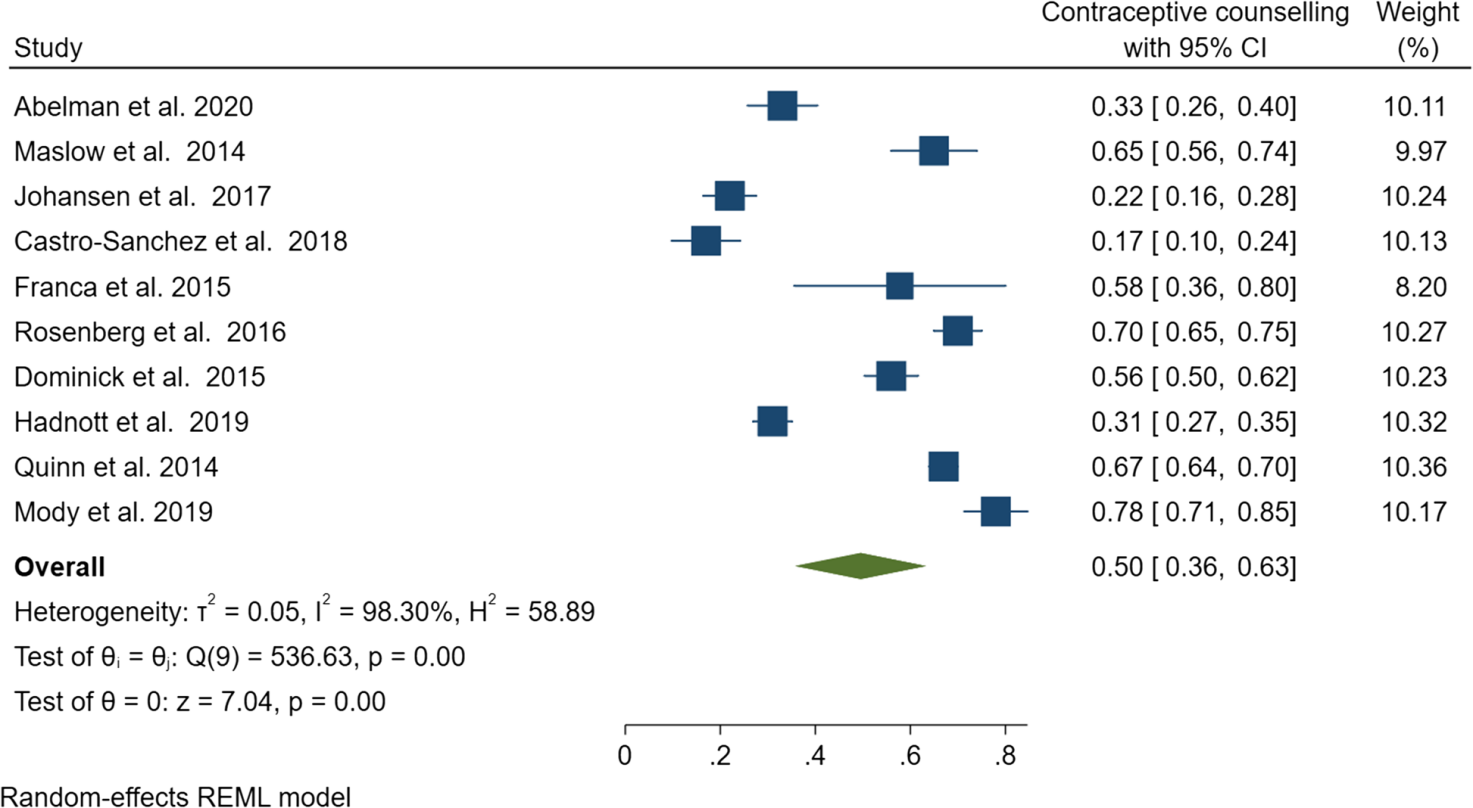
Pooled estimate of contraceptive counselling among women with cancer and cancer survivors

**Fig. 5:**
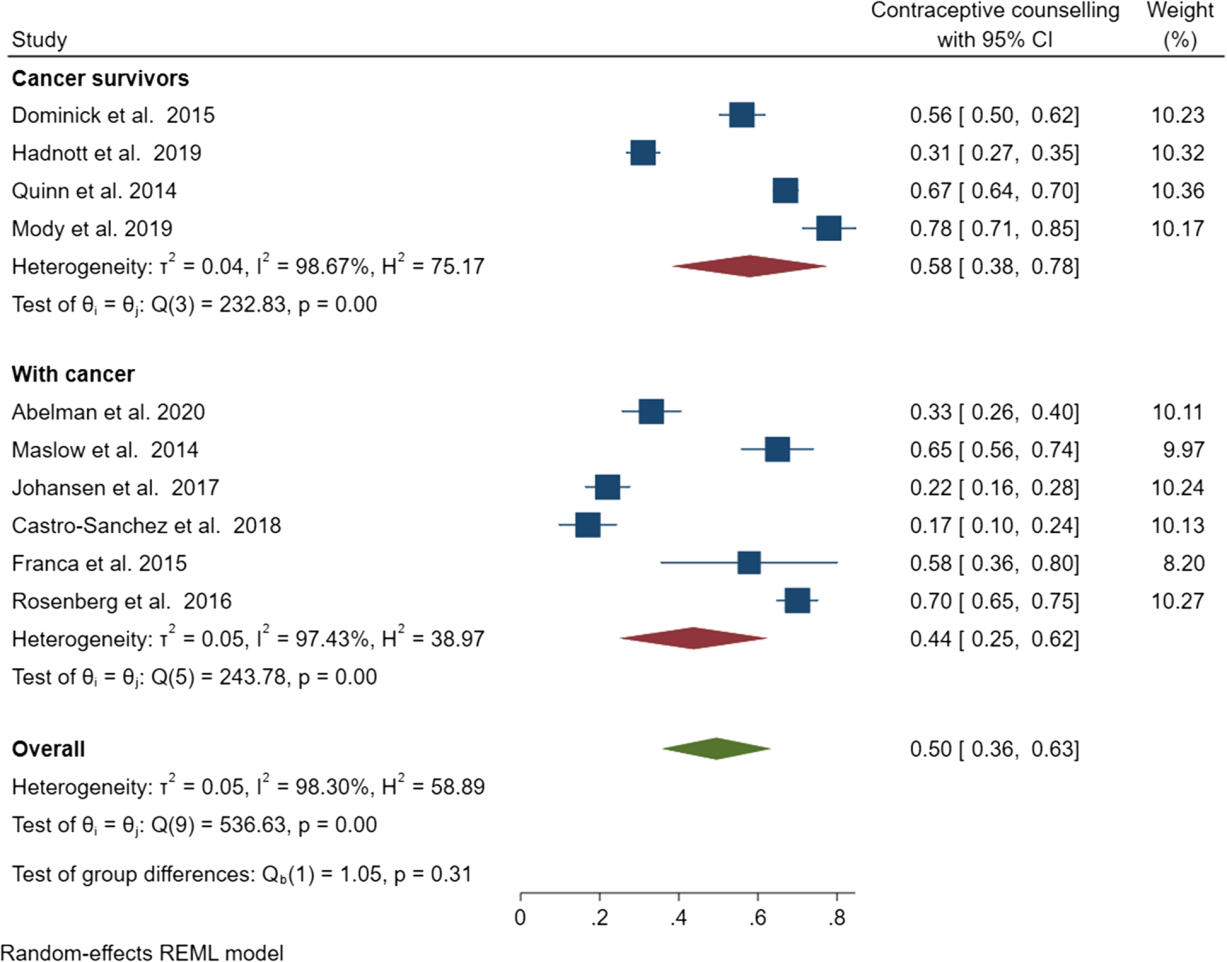
Subgroup analysis of contraceptive counselling by cancer status (women with cancer versus cancer survivors)

Among the ten quantitative studies involving women with cancer and cancer survivors, a pooled prevalence of 50% (95% CI: 36–63%) was found for engagement in contraceptive counselling. However, significant evidence of heterogeneity was noted (*Q* = 536.63, *ɽ*^2^ = 0.05, *I*^2^= 98.3%, *p* < 0.001) (Fig. 4). The pooled prevalence of contraceptive counselling was 44% (95% CI: 25–62%) (*Q* = 243.83, *I*^2^ = 97.4, *p* < 0.001) among women with cancer and 58% (95% CI: 38–78%) (*Q* = 232.83, *I*^2^ = 98.7, *p* < 0.001) for cancer survivors (Fig. 5). Given the heterogeneity among these studies, a narrative review is provided below.

One study assessed contraceptive counselling at 3-month intervals over 2 years among women newly diagnosed with breast cancer [45] and one retrospective study among cancer survivors included only women who discussed contraception [34]. Two studies from the USA reported on the relationship between age and provision of contraceptive counselling among women with cancer, although the findings were inconsistent. A retrospective study among young women with cancer found that women aged 15–20 years were less likely to receive contraceptive counselling compared to women aged 21–25 years (OR = 0.31, 95% CI: 0.14–0.70) [25], while a retrospective chart review study found that older women (aged 40–45 years) were significantly less likely to receive contraceptive counselling compared to younger women (aged 18–29 years) (OR = 0.2, CI: 0.1–1.0) [39].

The professions of contraceptive counselling providers were reported in seven studies [25, 26, 36, 42, 43, 49]. Contraceptive counsellors ranged from health educators and nurses to gynaecologists and haematologist-oncologists. A Moroccan cross-sectional study among women with breast cancer showed most contraceptive counselling was performed generally by doctors [41]. A US cross-sectional study among young breast cancer survivors found that contraceptive counselling was shared between surgeons (37%), oncologists (61%) and gynaecologists (53%). Surgeons and oncologists discussed contraception more often before cancer treatment, and gynaecologists discussed contraception both before and after breast cancer treatment (surgery, chemotherapy and/or radiation). Among breast cancer survivors who discussed contraception (*n* = 115, 78%), components of contraceptive counselling focused on cancer treatment during pregnancy could impact the baby (55%) and pregnancy could affect the risk of cancer recurrence (27%). While pregnancy prevention was discussed, the reason to avoid pregnancy was not explained in 18% of cases. Among those who were counselled, 32% (*n* = 17) received specific contraception recommendations from surgeons, 51% (*n* = 37) from oncologists and 61% (*n* = 22) from gynaecologists. Among these health care providers, women were more likely to accept contraceptive recommendations from a gynaecologist (84%) or oncologists (53%). No women selected contraceptive methods based on the advice from surgeons. Participants reported that safety concerns had the biggest influence on their contraception method choice. These largely focused on the perceived risks associated with the copper IUD including the risk of infection (33%) and impact on fertility (21%) [26]. A Turkish qualitative study (*n* = 20) also showed that premenopausal women with breast cancer reported insufficient counselling on contraception from oncology staff, despite their willingness to receive such information [49].

Documented contraceptive change based on routine follow-up counselling among eligible reproductive-aged cancer survivors was assessed in one retrospective study [34]. Tier III barrier methods were found to be the most likely contraceptive method to be adopted (21%). Among the 96 breast cancer patients with an absolute contraindication to hormonal contraception, only three chose the tier I copper IUD following counselling, with the remainder choosing tier III condoms. Among the remaining women who discussed hormonal and non-hormonal methods (*n* = 195), 52.4% chose the combined oral contraceptive pill, while the vaginal ring was the preferred choice among 28.1% of women because it was perceived as effective but required less action than a daily pill. This method was largely chosen by survivors of blood cancers. The progestogen-only pill was chosen by 22.7% of women, and only 3.2% requested a tier I LARC. The remaining 44.3% of women opted for tier III barrier methods. For women opting for non-hormonal contraception, half of the women chose these methods due to a fear of hormones [34].

### Contraceptive counselling interventions to improve contraceptive use

Five studies evaluated the impact of contraceptive counselling on contraceptive use among women with cancer. In three studies, contraceptive counselling was found to significantly improve contraceptive use in general [18, 25, 47], where improvement is defined as both the increased uptake of any contraceptive method and the uptake of, or switching to, highly effective contraception. An RCT among young women with breast cancer and their oncologists in the USA found contraceptive counselling increased contraceptive use by twofold (OR = 2.13; 95% CI: 1.20–3.78) [47]. In another US prospective cohort study, contraceptive use among cancer survivors was 30% higher among those who received contraceptive counselling compared to those who had not (adjusted RR = 1.28; 95% CI: 1.07–1.53) [18]. When the focus was on the use of emergency contraception, a US cross-sectional study found contraceptive use increased by threefold following contraceptive counselling (RR = 3.22, *p* < 0.01) [44]. A retrospective study in the USA among young women with cancer found contraceptive use to be 3.36 times more likely among those that received counselling compared to those that did not (95% CI: 1.35–8.34) [25]. A US cross-sectional study also showed that women who received contraceptive counselling were almost seven times more likely to use highly effective contraception compared to women who did not (adjusted OR = 6.92; 95% CI: 1.14–42.11) [43]. In addition, a US retrospective study reported that women who were already using contraception were more likely to receive counselling [39].

Specific contraceptive method change following consultation with providers was documented in five studies; however, the effect of contraceptive change varied depending on the study type, the point along the cancer continuum that counselling was received (and by whom) as well as patient contraceptive use status prior to diagnosis. A prospective cohort study in the USA found cancer survivors who used tier I/II methods were more likely to have received family planning services compared to users of tier III/IV methods [18]. Furthermore, contraceptive counselling did not always result in a change to more efficacious contraception. A Moroccan cross-sectional study among women with breast cancer showed 67.3% were counselled about contraceptive methods at diagnosis, 17.6% at the consultation of surgery, 29.4% before starting chemotherapy, 35.3% during chemotherapy and 17.6% at the end of treatment. Prior to diagnosis, most women were users of tier II oral contraception pill (93%). However, after cancer diagnosis and contraceptive counselling, 50% of women were users of condoms (tier III), 18.6% withdrawal (tier IV) and 14.3% tubal ligation (tier I) [41].

## Discussion

This timely systematic review and meta-analysis has found a lack of high-quality research focused on contraceptive use and contraceptive counselling interventions among women with cancer. A pooled prevalence found that only 64% of women who have experienced cancer were users of contraception. When individual studies were assessed, it was found that women who have experienced cancer were more likely to use low-efficacy contraception or be non-users of contraception compared to the general population. While this suggests a clear need for contraceptive counselling interventions, our review also showed that there is a lack of focus on improving contraceptive uptake among this population, with the prevalence of contraceptive counselling differing between studies. Studies varied widely depending on the type of cancer, data source and the point along the cancer care continuum contraceptive counselling was provided. A pooled prevalence revealed that only 50% of women received contraceptive counselling. When contraceptive counselling was implemented, it was found to be effective in increasing contraceptive use. However, there was some bias with women being more likely to receive contraceptive counselling if they were already contraceptive users. Beneficial outcomes of contraceptive counselling were also found to be variable.

Despite the lack of high-quality studies, the finding that women with cancer have suboptimal contraceptive practices at all stages along the cancer care continuum is concerning. Non-use of contraception or use of low-efficacy contraception with high typical failure rates (e.g. condoms, withdrawal and fertility-based awareness methods) places these women at high risk of unintended pregnancy. Such practices have also been found among women of reproductive age with other health conditions, including in a substantial number of women with unintended pregnancy histories [50–52]. Although there is a lack of information on the overall prevalence of unintended pregnancy and abortion among women with cancer, women who have experienced cancer (including survivors of childhood cancer) have been found to have similar or higher rates of abortion compared to the general population and age-matched controls; however, specific rates are dependent on the cancer type [2, 18]. An Australian data linkage study found that around half of pregnancies that occurred 2 years following a breast cancer diagnosis were terminated, with a large proportion of abortions occurring in the first 6 months following diagnosis and when undergoing active treatment [53]. When experienced during treatment, unintended pregnancy places a significant burden on women, potentially impacting not only treatment options but also foetal outcomes [54]. Cytotoxic chemotherapy during pregnancy, particularly in the first trimester, is associated with an increased risk of congenital malformations [55]. Likewise, adverse maternal outcomes including caesarean delivery, gestational hypertension and in rare cases pregnancy-induced cardiomyopathy as well pregnancy outcomes such as pregnancy loss, preterm birth and low birth weight have been reported among childhood cancer survivors [56–58]. As such, access to high-quality contraceptive counselling within oncology settings and long-term follow-up through primary care are critical to optimise long-term cancer and reproductive health outcomes for all women who have experienced cancer, regardless of when in the life course it was experienced. Yet, this review has found that contraceptive counselling is inadequate and haphazard in its implementation.

Our study found that few studies directly addressed contraceptive change (including reasons for change) across the cancer care continuum. Only one study reported on an RCT (although this was in abstract form), and only one cross-sectional study examined contraceptive use at the time of diagnosis, during treatment and post-treatment [26, 47]. When aspects of received contraceptive counselling were reported by women, they indicated that the quality was poor with reasons to avoid pregnancy not adequately explained. Some women reported being informed of the risks of cancer recurrence and potential impact on the foetus, while others indicated that there was limited information on the impact of cancer apart from discontinuing hormonal contraception and changing to non-hormonal methods [26, 48]. Bias in the delivery of contraceptive counselling interventions was also noted across the available studies. A US retrospective chart review study found that patients had a threefold increase in the receipt of contraceptive counselling if they were currently using a method of contraception at diagnosis. Contraceptive counselling was also less likely to be provided to women of older reproductive age [59]. Lower contraceptive use at older reproductive age has been found in the general population and among women with chronic diseases across the reproductive life course [60, 61]. Excluding the specific impact of cancer and its treatment on women, pregnancies over the age of 40 carry significant maternal and neonatal risks including increased risk of chromosomal abnormalities, miscarriage and premature delivery as compared to younger women. As such, guidelines indicate that women over the age of 40 years should use effective contraception until after menopause to prevent unintended pregnancies [62, 63].

Health care providers are the gatekeepers of contraceptive knowledge particularly where health conditions are concerned. Yet, these findings point to unmet informational needs regarding evidence-based contraceptive advice and support for women with cancer across the reproductive life course, even when contraceptive counselling is indicated. Previous research has indicated that when asked about contraceptive screening and referral practices, health providers described conducting other forms of counselling or provided pregnancy screening in place of comprehensive and directed contraceptive counselling [27, 64]. Furthermore, some providers described counselling to specifically avoid pregnancy without offering contraceptive counselling or referral to qualified specialists such as a gynaecologist. Meanwhile, others counselled women to avoid sex for certain indications such as infection during periods of neutropenia only or to prophylactically address issues around heavy bleeding [27, 36]. As such, the source of contraception may play a key role in not only determining if contraceptive counselling is provided but the focus of the counselling. It has been suggested that women prefer to receive their contraceptive counselling from oncologists. However, it has been reported that although oncologists view contraceptive use as important in cancer surveillance, few provide recommendations, even when explicitly asked by patients [14, 26]. Others, however, have posited that the dearth of contraceptive counselling is attributed to a lack of clear responsibility among oncology providers, communication issues between team members and other specialists as well as clinician-perceived lack of formalised medical education and training [27]. While beyond the scope of this review, in order to design effective contraceptive counselling interventions, an in-depth understanding of the extent and nature of the barriers to its implementation and its impact on patient care is required.

While the findings report a lack of standardised contraceptive care for women with cancer, in general, women with breast cancer were found to use effective contraception at lower rates than women with non-breast malignancies, despite similar overall rates of contraceptive use [43]. This gap in the provision of high-efficacy contraception may be attributed to the recommendations around the use of hormonal contraception. The UK Medical Eligibility guidelines do not recommend the use of hormonal-based methods in women with current breast cancer as they present an unacceptable health risk (category 4) [22]. For women with a history of breast cancer, such methods are not recommended with the risks of the method outweighing the advantages (category 3). Therefore, given that hormonal methods such as the combined oral contraceptive pill are the most prevalent method of contraception across the reproductive life course [61, 65], these women require appropriate advice regarding the efficacy of available non-hormonal methods. Although the copper IUD is regarded as a first-line contraceptive method for women with hormone-dependent cancer, the uptake of this method was found to be low. This indicates that significant barriers to its uptake exist. While studies on the barriers to the copper IUD among breast cancer patients are limited, it has been found that women have concerns about pain during placement (which echoes concerns about LARC in the general population) and potential infection risks [26, 66]. This is despite guidelines recommending their use among immunocompromised women, including those with cancer.

Therefore, given a lack of effective contraceptive use (including LARC) was noted across most cancer types and points along the cancer care continuum (e.g. at diagnosis and survivorship), understanding patient-related barriers to their uptake is required. It is generally understood that a driving factor may be related to misperceptions surrounding fertility [23, 33, 38]. Although chemotherapy-induced amenorrhea is common in women with cancer, even when exposed to gonadotoxic treatment, the impact on ovarian function varies widely, with a large portion of women maintaining reproductive function [67]. Importantly, a large survey of premenopausal women with invasive breast cancer found that more than 85% of women reported resumption of menstruation 12 months after completion of chemotherapy, with the majority returning within 6 months [68]. This suggests that a lack of menses is a poor marker of infertility in this population. However, Guth and colleagues found 16% of oncologists assessed contraceptive use in their patients only if menses resumed [14]. While women in the general population choose their method of contraception for a number of reasons (and effectiveness being only one of them) [69], apart from breast and gynaecological cancers, other cancer types have no contraindications to hormonal contraception following cessation of treatment, except where residual cardiotoxicity and the presence of any other comorbidities and complications exist [22]. An Italian retrospective chart review study found that hormonal contraception was unsuitable in only four cancer survivors due to medical or oncology-related contraindications, yet a large proportion of patients still refused these methods following counselling [34]. On the other hand, despite the existence of clinical medical eligibility guidelines, this review found the inappropriate selection of hormonal contraceptive methods among women with breast cancer [47]. This finding further underscores the importance of targeted shared decision-making in counselling women with cancer based on their specific form of cancer.

A key strength of this review is that we used a comprehensive search for the best possible evidence currently available. Despite this, our review has some limitations. Included studies were heterogeneous in terms of study type, setting and population. There was a lack of clarity among studies in relation to the contraceptive counselling provided. Some studies were associated with small size and sampling bias as they were carried out using chart reviews or provided abstracts only. The available studies were largely from the US and primarily focused on breast cancer; most studies did not examine contraceptive use and change by cancer type, and there was a lack of population-level studies. Finally, no studies reported on the long-term benefits and effects of contraceptive counselling and contraceptive use among women with cancer.

## Conclusions

This review found an alarming lack of high-quality research concerned with contraceptive counselling in the context of cancer diagnosis and treatment. Where studies had been conducted, the methods were variable, and the results of contraception counselling were equivocal. Despite this, the findings showed low contraception uptake generally, and high uptake of low-efficacy contraception particularly among women with cancer. This indicates an urgent unmet need for contraception counselling in this population. Although fertility preservation is important for young women with cancer, it should not be the focus to the exclusion of contraceptive counselling. Given the impact associated with unintended pregnancy, particularly among this vulnerable population, contraceptive counselling interventions tailored to specific cancer types, disease stage, comorbidity, reproductive goals and preferences are required. Such interventions should address the patient experience and reasons for contraceptive choice as well as the concerns and misperceptions around hormonal contraception and LARC (including the copper IUD where breast cancer is concerned). There is also a need for high-quality studies (e.g. randomised controlled trials) that evaluate the uptake of, compliance with, and satisfaction of structured contraceptive counselling in oncology settings as well as rapid access pathways to LARC insertion prior to treatment initiation for women choosing this method. The results of this review also highlight the adjacent need for high-quality, generalisable longitudinal research to identify the predictors of effective contraception uptake in the context of cancer and the impact of different types of counselling on such uptake, to ensure the best possible outcomes for women with cancer.

## Supplementary Information

**Additional file 1: Table S1.** Summary of study inclusion and exclusion criteria. **Table S2.** Example search strategy in Ovid Medline. **Table S3.** Characteristics of included studies.

## Data Availability

Not applicable.

## Abbreviations

CINAHL: Cumulative Index to Nursing and Allied Health Literature
IUD: Intrauterine device
LARC: Long-acting reversible contraception
OR: Odds ratio
PRISMA: Preferred Reporting Items for Systematic Reviews and Meta-Analysis guidelines
RCT: Randomised controlled trial
RR: Relative risk
