# Supplementary material for: Contraceptive use and contraceptive counselling interventions for women of reproductive age with cancer: a systematic review and meta-analysis
Source: BMC Med. 2022 Dec 17;20:489. doi: 10.1186/s12916-022-02690-w (PMC9759910; doi:10.1186/s12916-022-02690-w)
Supplement: Supplementary file 1 — Additional file 1: Table S1. Summary of study inclusion and exclusion criteria. Table S2. Example search strategy in Ovid Medline. Table S3. Characteristics of included studies. [file 12916_2022_2690_MOESM1_ESM.docx]

**Additional file 1**

**Table S1. Summary of study inclusion and exclusion criteria.**

| **Inclusion** | **Exclusion** |
| --- | --- |
| All malignancies diagnosed in females when aged 15-49 years | Studies focused on men (where women of reproductive age could not be disaggregated) and cancers diagnosed outside the reproductive period |
| Contraceptive counselling experienced after a cancer diagnosis (e.g., phone and face-to-face) involving trained healthcare providers | Contraceptive counselling prior to a cancer diagnosis |
| All contraceptive methods including emergency contraception | Contraception examined as a risk factor for cancer development |
| Studies focused on contraceptive uptake, continuation, or method switching | Studies on contraceptive use where women with cancer could not be disaggregated from women with other health conditions |
| Peer-reviewed articles with cross-sectional, case-control, cohort, implementation, clinical trials, and qualitative study designs | Case-reports, clinical guidelines, reviews and position papers, clinician perspectives |
| English | All other languages |

**Table S2. Example search strategy in Ovid Medline.**

| **Search ID #** | **Search terms** | **Results** |
| --- | --- | --- |
| 1 | (counsel?ing or selection tool*).ti,ab. | 102168 |
| 2 | (education* adj (strateg* or program* or intervention*)).ti,ab. | 62501 |
| 3 | (decision* adj (tool* or aid*)).ti,ab. | 4539 |
| 4 | (quality adj4 (family planning or contracepti*)).ti,ab. | 740 |
| 5 | (patient adj3 (provider interaction* or provider communication*)).ti,ab. | 1885 |
| 6 | (client adj3 (provider interaction* or provider communication*)).ti,ab. | 107 |
| 7 | (("birth control" or contracepti* or "family planning" or "fertility regulation") adj4 (counsel* or decision-making or "decision support" or "decision aid*" or "decision tool" or "decision tools" or educat* or "informed choice*" or select* or shared-decision* or "tiered-effectiveness")).ti,ab. | 5745 |
| 8 | ((family planning or contracept*) adj4 (intervention* or implement* or therap*)).ti,ab. | 3130 |
| 9 | 1 or 2 or 3 or 4 or 5 or 6 or 7 or 8 | 174784 |
| 10 | (birth control or fertility regulation or contracepti* or family planning or birth control or depo?-medroxyprogesterone or depo? Medroxyprogesterone or Depo-Provera or IU?D or IUD or IUDS or IUS or intra?uterine device* or intra?uterine system* or implant* or implanon or jadelle or norplant*).ti,ab. | 517603 |
| 11 | exp Contraception/ or Contraceptive Agents/ or exp Contraceptive Agents, Female/ or Contraception Behavior/ or Contraceptive Devices/ or exp Contraceptive Devices, Female/ or Family Planning Services/ or *LONG-ACTING REVERSIBLE CONTRACEPTION/ | 117312 |
| 12 | (("birth control" or contracepti* or "family planning" or "fertility regulation") adj4 (continu* or discontinu* or uptake or initiat* or switch* or satisf* or using or use*)).ti,ab. | 31811 |
| 13 | 10 or 11 or 12 | 572567 |
| 14 | (cancer* or tumo?r* or neoplas* or malignan* or carcinoma* or adenocarcinoma* or choriocarcinoma* or leuk?emia* or metastat* or sarcoma* or teratoma*).ti,ab. | 3763883 |
| 15 | exp Chemotherapy, cancer/ or exp radiotherapy/ | 198817 |
| 16 | (Chemotherapy, cancer* or Radiotherapy).ti,ab. | 181026 |
| 17 | 14 or 15 or 16 | 3846688 |
| 18 | 9 and 13 and 17 | 1198 |
| 19 | limit 18 to (human and English language) | 880 |

**Table S3. Characteristics of included studies.**

| **Author and year** | **Country** | **Design and setting** | **Participant characteristics** | **Cancer type and treatment received** | **Contraceptive counselling (Prevalence and source)** | **Contraceptive use (Prevalence and type)** | **Other findings and study limitations** |
| --- | --- | --- | --- | --- | --- | --- | --- |
| Abelman et al. 2020 [25] | USA | Retrospective chart review; Yale New Haven Hospital | 157 adolescents and young adults with cancer; aged 15-25 years (mean=20.5 years) | *Type:* Haematologic (40.8%); thyroid (31.2%); neurologic (9.6%); sarcoma (5.1%); breast (5.7%); gynaecologic (5.1%)  *Treatment:* Chemotherapy (60.5%); surgery (52.2%), radiation (52.2%); targeted/biologic treatments (17.8%); bone marrow transplant (5.1%); hormone therapy (4.5%) | *Prevalence:* 33.1% had documented counselling in medical records during treatment.  *Source:* Oncologist | *Prevalence:* 48.4% had documented contraceptive use during cancer treatment  *Specific methods:* Combined oral contraceptive pill (24.2%); condoms (7%); depo injection (9.5%); progestogen-only implant (1.3%); IUD (4.5%); other (1.9%); no method (51.6%) | *Limitations:* Potential missing data due to retrospective chart review; around 30% had missing sexual activity status; did not distinguish between progestogen and copper IUD use; did not assess method-specific contraceptive change due to counselling |
| Castro-Sanchez et al. 2018 [35] | Mexico | Cross-sectional self-report survey; large tertiary health care facility in Mexico City | 104 women with cancer aged 18-40 years (median=34) who completed chemotherapy in the last 5 years or who were currently receiving long-term treatment with hormonal therapy and/or trastuzumab | *Type:* Breast cancer (100%)  *Treatment:* Chemotherapy (88.5%); hormone therapy/treatment with trastuzumab (67.3%) | *Prevalence:* 16.7% advised about using contraception during treatment  *Source:* Health care providers | *Prevalence:* 51.1% used contraception during chemotherapy; 45.7% used contraception while receiving hormonal therapy and/or treatment with trastuzumab  *Specific methods:* Sterilisation (9.6%); IUDs (9.6%); oral hormonal contraception (1.0%); condom (21.2%); withdrawal (4.8%); rhythm (2.0%); abstinence (2.0%); no method (49.0%) | *Limitations:* Retrospective; cross-sectional; small sample size; did not distinguish between progestogen and copper IUD use; did not assess method-specific contraceptive change due to counselling |
| Cutler et al. 2016 [36] | USA | Retrospective chart review at University of  California, Davis Medical Centre | 137 women with cancer aged 14-40 years (mean=29 ± 8 years) | *Type:* Leukemia; lymphoma; malignant solid tumours  *Treatment:* Chemotherapy (100%) | *Prevalence:* 18%  received menstrual suppression counselling prior to chemotherapy initiation to prophylactically address heavy  menstrual bleeding as a complication of thrombocytopenia  *Source:* Haematologist-oncologists | *Prevalence:* 71% (17/24) counselled prior to chemotherapy started prophylactic menstrual suppression  *Specific methods among contraceptive users:* Combined oral contraception (n=13); depot medroxyprogesterone acetate (n=2) | *Limitations:* Focused on contraceptive counselling for the purpose of menstrual bleeding; single site; retrospective review; reliant on clinician documentation and coding issues; no documentation of pregnancy risk |
| Dominick et al. 2015 [18] | USA | National prospective cohort study on reproductive health after cancer (Fertility Information Research Study) | 295 cancer survivors aged 20-44 years (mean=31.6 ± 5.7 years) at risk of pregnancy | *Type:* Breast (31.5%); lymphoma (24.5%); gynaecologic (9.7%); blood (5.2%); thyroid (5.2%); other (21.5%)  *Treatment:* Chemotherapy (79.6%); surgery (63.3%); radiation (48.4%); bone marrow or stem cell transplant (5.5%) | *Prevalence:* 56% received family planning services since  cancer diagnosis; 50% reported receiving them in past 12 months.  *Source:* Not reported | *Prevalence:* 57.4% of survivors used contraception compared with 68.6% in the general population. Survivors were less likely to use  tiers I–II methods compared with the general population (34.2% vs 53.0%)  *Specific methods:* Sterilisation (4%); IUD (13%); combined oral contraceptive pill (37%); condom (36%); contraceptive patch (1%); contraceptive ring (5%); depo injection (1%); withdrawal (2%); periodic abstinence (1%); emergency contraception (10%) | *Limitations:* Small sample size,; self-reported study variables; inability to determine causation with the cross-sectional design; didn’t assess contraceptive use by cancer type |
| Franca et al. [37] | Brazil | Cross-sectional survey; outpatient clinic | 19 adolescent and young adults with cancer aged 10-19 (mean = 15 years ± 2.4 years) | *Type:* All cancers but most had osteo-sarcoma (42%) or leukaemia (36%)  *Treatment:* Not reported | *Prevalence:* 57.8%  *Source:* Not reported | *Prevalence:* Not reported  *Specific methods:* Among those who were sexually active, the most frequently used methods were oral or  injectable hormonal contraceptives (89%) and condoms (15%). 21% had never used any contraceptive method | *Limitations:* Single institution; very small sample; cross-sectional; abstract only |
| Guth et al. 2016 [38] | Switzerland | Prospective cohort survey (Basel Breast Cancer Database) | 100 women with cancer aged 26-40 years (mean=36.5 years) at initial diagnosis | *Type:* Breast cancer (100%)  *Treatment:* Radiotherapy and chemotherapy |  | *Prevalence:* Not reported  *Specific methods:* 42% were either not at risk of an unintended pregnancy or used Tier 1 contraception including sterilisation (12%) or Copper IUD (4%). No contraception or ineffective tier III/IV contraception were used by 34%; 8% used condoms; 16% were using hormonal contraception which needed to be stopped | *Limitations*: Retrospective study design; small sample; limited to the first year after diagnosis |
| Hadnott et al. 2019 [23] | USA | Cross-sectional online survey (Reproductive Window  Study); state cancer registries, physician referrals | 483 cancer survivors aged 18-40 years (median=34 years) | *Type:* Breast (23.4%); leukemia/lymphoma (31.7%); gynaecologic (cervix, uterus, ovary) (5.8%); gastrointestinal (pancreas, gallbladder, stomach, small intestine, colon, appendix, rectum) (2.9%); bone/soft tissue (6.8%); thyroid/skin (29.4%)  *Treatment:* Chemotherapy (62.9%); bone marrow transplant (3.1%); surgical cancer therapy (68.7%); radiation (48.7%) | *Prevalence:* 31% received family planning counselling within 12 months of participation  *Source:* Not reported | *Prevalence:* 84%; 36.0% were classified as Tier I; 23.2% as Tier II; 29.1% as Tier III; 10.8% as Tier IV  *Contraceptive use by cancer type:* Breast (22.9%); leukemia/lymphoma (30.8%); gynaecologic (5.4%); gastrointestinal (2.7%); bone/soft tissue (7.1%); thyroid/skin (31.0%)  *Specific methods:*  Barrier/withdrawal (49.8%); combined hormonal contraceptives (22.7%); IUD (20.7%); 14.8% sterilisation; rhythm method (1.5%); Depo-Provera (1.5%), and contraceptive implant (1.2%). | *Limitations:* Cross-sectional; did not separately report hormonal vs. copper IUD. |
| Harries et al. 2020 [48] | South Africa | Qualitative interview study; tertiary hospital | 24 women with cancer aged 18-49 years (median=36.5 years) | *Type:* Breast cancer (100%)  *Treatment:* Not reported |  | *Prevalence:* 70% since diagnosis.  *Specific methods:* Among those using contraception, half used the Copper IUD; other methods included sterilisation and condoms | *Limitations:* Small sample; single site clinical sample |
| Johansen et al. 2017 [39] | USA | Retrospective chart review | 211 women with cancer undergoing chemotherapy aged 18-45 years (median=40 years) | *Type:* Breast cancer (100%)  *Treatment:* Chemotherapy (100%) | *Prevalence:* 22% received contraceptive counselling; 10% received contraceptive counselling alone and 12% received both contraceptive and fertility preservation counselling.  *Source:* Not reported | *Prevalence:* Current contraceptive (25%); past contraception (74%).  *Specific methods:* Not reported | *Limitations:* Chart review with a relatively small sample size; lack of diversity |
| Karaoz et.al. 2010 [49] | Turkey | Qualitative interviews at oncology clinic | 20 women with cancer aged 21-50 years (mean=40.2 years) | *Type:* Breast cancer (100%)  *Treatment:* Mastectomy (100%); lymph node excision 65%); chemotherapy (85%); radiation therapy (45%); tamoxifen (50%) | *Prevalence:* 40% (n=8) received contraception counselling after diagnosis.  *Source:* 6/8 received information from nurses and 2/8 received counselling from physicians. | *Prevalence:* Not reported  *Specific methods:* Withdrawal or none (75%); IUD (10%); tubal sterilisation (15%) | *Limitations:* Small sample; single site |
| Knight et al. 2014 [40] | Not stated | Retrospective record review (using ICD 9 codes) | 112 women with cancer aged 18-45 years | *Type:* Non-gynaecologic cancers  *Treatment:* Chemotherapy; radiation |  | *Prevalence:* 45% had documentation of contraceptive method  prior to initiation of treatment  *Specific methods:* Not reported | *Limitations:* Abstract only |
| Lakhdissi et al. 2017 [41] | Morocco | Cross-sectional survey at National Institute of Oncology Rabat | 72 women of reproductive age (mean=41.4 ± 6.3 years) | *Type:* Breast cancer (100%)  *Treatment:* 97.2 % were under treatment; 17.9% had consultation for surgery; 29.4% received chemotherapy | *Prevalence:* 52% were informed that hormonal contraception was not recommended; 67.3% were informed at diagnosis, 17.6% at surgery consultation, 29.4% before chemotherapy initiation, 35.3% during chemotherapy and 17.6% at the end of treatment.  *Source:* 86.5% received information from doctors. | *Prevalence:* 98.2% prior to diagnosis; 75% after diagnosis  *Specific methods prior to diagnosis:* Oral contraception (93%); IUD (5.6%); and condom (1.4%)  *Specific methods following diagnosis:* Oral contraception (24.1%); IUD (18.4%); barrier methods (55.6%); tubal sterilisation (1.9%) | *Limitations:* Abstract only. |
| Madrigal et al. 2019 [42] | USA | Pilot intervention study of referrals to family planning department from oncology department | 36 women with cancer aged 17-47 years (mean=34.1 years) | *Type:* Breast (75%); other cancer (25%)  *Treatment:* Not reported | *Prevalence:* 28.6% of women who had not completed childbearing and presented for fertility preservation were  counselling before beginning cancer treatment.  *Source:* Nurse; medical assistant; medical student; health educator | *Prevalence:* Not reported  *Specific methods following counselling:* Of those sexually active, 45.4% chose reversible long-term contraception, and 36.4% chose short-term contraception at initial visit; 52.8% used Tier I; 11.1% used Tier II and 36.1% used Tier III methods | *Limitations:* Pilot study; small sample |
| Maslow et al. 2014 [43] | USA | Cross-sectional survey; large tertiary health system | 107 women with cancer aged 18-45 years | *Type:* Breast (52%); lymphoma (22%); leukemia (8%); cervical (2%); ovarian (3%) sarcoma (3%); other (9%)  *Treatment:* Not reported | *Prevalence:* 65% prior to treatment initiation  *Source:* Health care professional | *Prevalence:* 77% used contraception during cancer treatment; 5% used Tier I methods; 23% used Tier II methods; 31% used Tier III methods; 4% used Tier 4 methods  *Specific methods:* IUD (4%); partner vasectomy (1%); combined oral contraceptive pill (21%); nuva-ring (1%); diaphragm (1%); condoms (21%); withdrawal (10%); rhythm method (2%); spermicidal jelly (1%); body temperature (1%); abstinence (25%); no method (14%) | *Limitations:* Potential recall bias; did not examine multiple method use; did not explore the content and degree to which participants were counselled; potential sampling bias – all women were well-educated and had access to tertiary medical care and internet-based technologies at a single institution |
| Massarotti et al. 2021 [34] | Not reported | Retrospective clinical record review; gynaecological department at a university tertiary hospital | 280 post-menarche cancer survivors aged 27-35 years (mean=32 years) | *Type:* Breast (33.9%); haematologic (28.2%); gynaecological (15%); gastroenterological (12.5%); other 10.4%)  *Treatment:* Surgery (70.7%); chemotherapy (85.7%); radiotherapy (54.3%); bone marrow transplant (8.2%); Hormonal therapy only (0.7%); highly gonadotoxic therapies (11.1%) | *Prevalence:* Counselling was described as part of routine follow-up care although 3.2% specifically requested contraceptive counselling at their follow-up visit.  *Source:* Physician | *Prevalence among breast cancer survivors:* Copper IUD or barrier methods were offered to breast cancer patients with an absolute contraindication to hormonal contraception (n=96); only 3.1% were interested in the copper IUD; the remainder (96.9%) indicated that they would use condoms  *Prevalence among other cancers (n=185):* Combined oral contraceptive pill (52.4%); vaginal ring (28.1%); progestogen-only pill (3.2%); patch (1.6%); LARC (3.2%) | *Limitations:* Single site study |
| McLean et al. 2014 [44] | USA | Retrospective cohort survey; Fertility Information Research Study  (FIRST) | 267 cancer survivors aged 20-44 years (mean=31.6 ± 5.7 years) | *Type:* All cancers; breast cancer was the most prevalent (33%)  *Treatment:* All had exposure to gonadotoxic therapies |  | *Prevalence:* 11%  used emergency contraception after cancer diagnosis | *Limitations:* Abstract only |
| Mody et al. 2019 [26] | USA | Cross-sectional online survey | 150 cancer survivors aged 18-50 years (mean=37 ± 6 years) | *Type:* Breast cancer (100%)  *Treatment:* Lumpectomy (29.3%); unilateral mastectomy (18.7%); bilateral mastectomy (48%); chemotherapy (78%); radiation (61.3%); hormone therapy (62%) | *Prevalence:* 78% discussed contraception.  *Source:* Surgeon (35.3% before and 1.3% after); oncologist (48.7% before and 12.7% after); gynaecologist (24% before and 29.3% after) | *Prevalence:* Among sexually active survivors who were not trying to become pregnant, 94% reported contraception before diagnosis; 90% during treatment; and 83% after treatment  *Specific methods before treatment:* Sterilisation (6.5%); hormonal or unknown IUD (8.9%); copper IUD (3.3%); pill, patch, ring or depo injection (52.9%); condoms (21.1%); withdrawal or rhythm method (0.8%); none (6.5%)  *Specific methods during treatment:* Sterilisation (11.4%); hormonal or unknown IUD (3.81%); copper IUD (10.5%); pill, patch, ring or depo injection (1.9%); condoms (52.4%); withdrawal or rhythm method (9.5%); none (10.5%)  *Specific methods after treatment:* Sterilisation (16.1%); hormonal or unknown IUD (2.4%); copper IUD (23.4%); pill, patch, ring or depo injection (1.6%); condoms (29%); withdrawal or rhythm method (10.5%); none (16.9%) | *Limitation:* Not representative; lack of diversity and generalisability; potential recall bias |
| Patel et al. 2015 [45] | USA | Prospective observational survey; public hospital | 11 women with cancer aged 18-49 years (mean=39 years) | *Type:* Breast cancer  *Treatment:* Medical oncology | *Prevalence:* All counselled | *Specific methods after counselling:* Permanent sterilisation (18%); IUD (55%); abstinence (18%); condoms (9%) | *Limitations:* Small sample; pilot study |
| Patel et al. 2009 [33] | USA | Cross-sectional survey (health assessment); oncology clinic | 20 women with cancer aged 15-44 years (mean = 36.6 years) | *Type:* Breast cancer (90%); other (10%)  *Treatment:* Modified radical mastectomy (83.33%); chemotherapy alone (55%); chemotherapy and radiotherapy (40%) |  | *Specific methods:* Abstinence was the preferred method by 54.6 % of the women. The remainder used barrier methods (condoms and foams). | *Limitations:* Pilot study; small sample; lack of detail regarding contraception |
| Quinn et al. 2014 [46] | USA | Retrospective survey data linked with California  Cancer Registry | 918 cancer survivors aged 18-40 years | *Type:* Non-gynaecological cancer  *Treatment:* Chemotherapy, radiation, or sterilising surgery | *Prevalence:* 66.7% were at risk of pregnancy and received pre-treatment counselling | *Prevalence:* 46.6%  *Specific methods:* Barrier (26%); hormonal (25%); tubal sterilisation (21%); vasectomy (18%); IUD (7%); other (4%); withdrawal (29.5%) | *Limitations:* Sampling bias, response bias |
| Rosenberg et al. 2016 [47] | Not reported | Randomised controlled trial; 54 sites | 312 women at risk of pregnancy aged 22-45 years (mean=39) | *Type:* Breast cancer (100%)  *Treatment:* Not reported | *Prevalence:* 70% | *Prevalence:* 92%  *Specific methods prior to diagnosis:* 39% used highly effective contraceptive methods  *Specific methods after diagnosis:* 52% reported current use of or a plan to use a highly effective method; 6% reported use of a hormonal birth; 2% reported withdrawal as their only contraceptive method; 8% reported no contraception. | *Limitations:* Abstract only |
